# Supplementary material for: Interband resonant high-harmonic generation by valley polarized electron–hole pairs
Source: Nat Commun. 2019 Aug 16;10:3709. doi: 10.1038/s41467-019-11697-6 (PMC6697745; doi:10.1038/s41467-019-11697-6)
Supplement: Supplementary file 1 — Supplementary Information [file 41467_2019_11697_MOESM1_ESM.pdf]

Supplementary Information

Interband resonant high-harmonic generation by valley polarized electron-hole pairs

Yoshikawa et al.

I. Supplementary figures

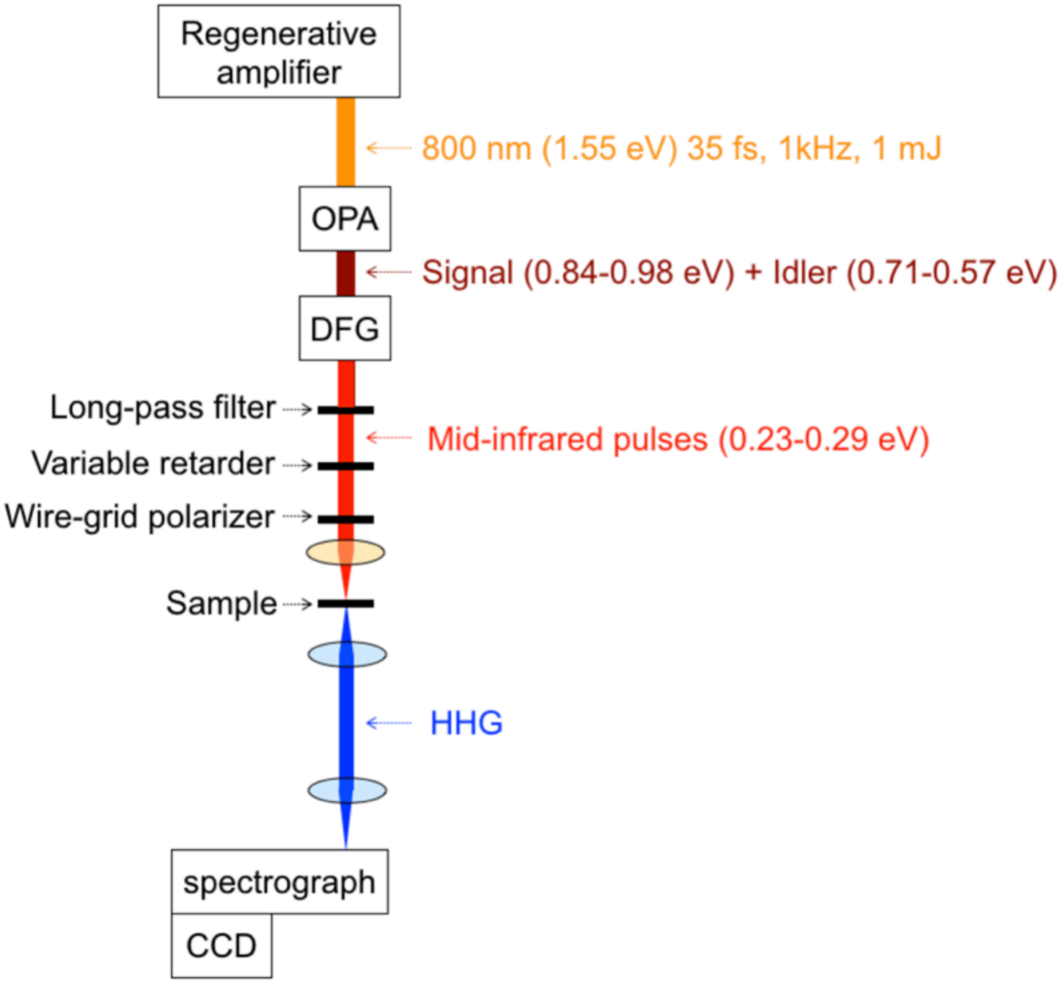

Supplementary Figure 1 Schematic diagram of the experimental setup. The experimental setup for the HHG measurements.

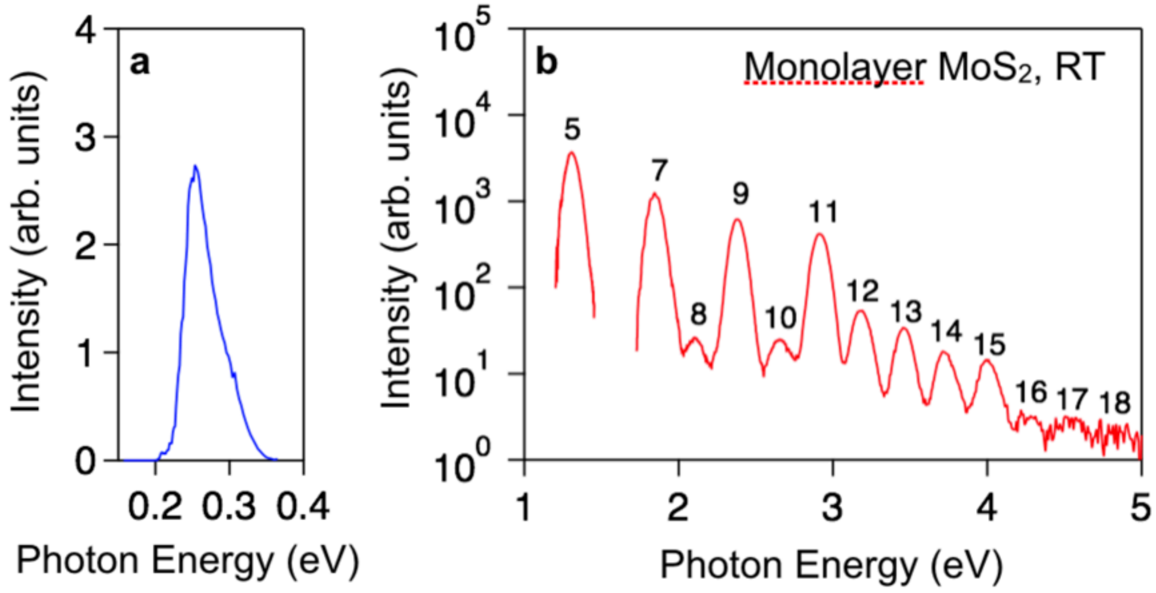

Supplementary Figure 2 The spectrum of mid-infrared pump pulse and HHG spectrum up to 18th order. Spectrum of **a** mid-infrared excitation light and **b** HHG emitted from monolayer MoS_2.

II. Supplementary Note 1: Polarization of high-harmonic generation (HHG) from monolayer MoS_2

The CVD samples had many crystal domains of monolayers, and their crystal orientations of the monolayer islands are not identical. We measured the harmonic spectra at the position on the sample where strong even-order high harmonics were observed; that is, the crystal orientation with respect to the polarization of the incident laser was optimized to maximize the even-order harmonics. Supplementary Figure 3 shows the polarization of the ninth and twelfth harmonics with the zigzag and armchair excitation. The odd-order harmonics are always parallel to the incident light polarization. The even-order harmonics with the zigzag excitation, where the even-order harmonics are generated efficiently, are perpendicularly polarized to the excitation light, while those with the armchair excitation are parallel. These polarization selection rules are originated from the dynamical symmetry of TMDs under the strong driving field and are discussed using the microscopic HHG model in solids in the Supplementary Note 4.

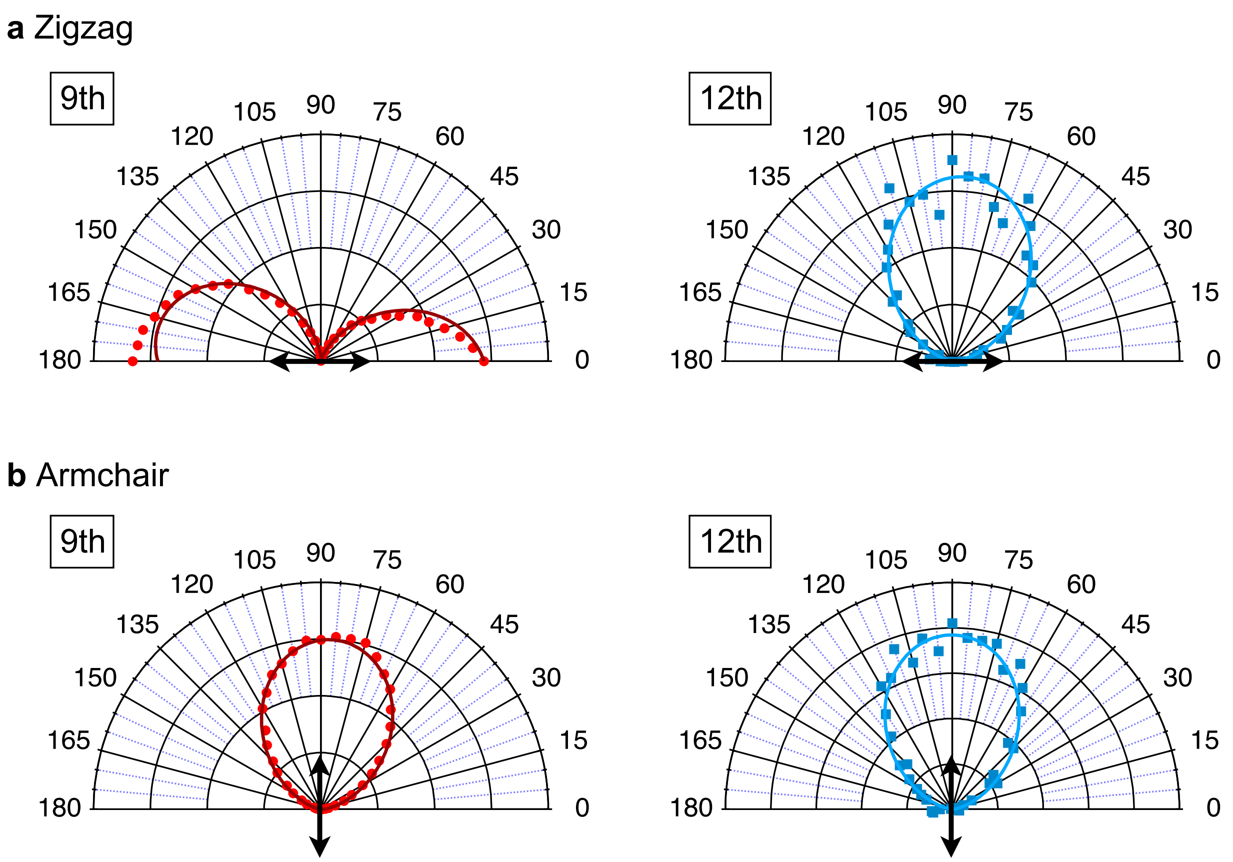

Supplementary Figure 3 Polarization of HHG from monolayer MoS_2. Polarization of ninth and twelfth harmonics under **a** zigzag and **b** armchair excitations in monolayer MoS_2. The black arrows indicate the polarization of the incident mid-infrared light.

III. Supplementary Note 2: Nonperturbative behavior of HHG in monolayer MoS_2

Supplementary Figure 4 shows the intensity of the high harmonics generated from monolayer MoS_2 as a function of the peak power of the excitation pulses $I_{exc.}$. The power dependence shows a saturation-like behavior, whereas it should show an I^n dependence (n means harmonic order) in the perturbative limit. All of the observed, both even and odd, harmonics show an $\sim I^3$ dependence at the highest excitation power used in this study. The power dependence confirms the non-perturbative behavior of HHG in monolayer TMDs.

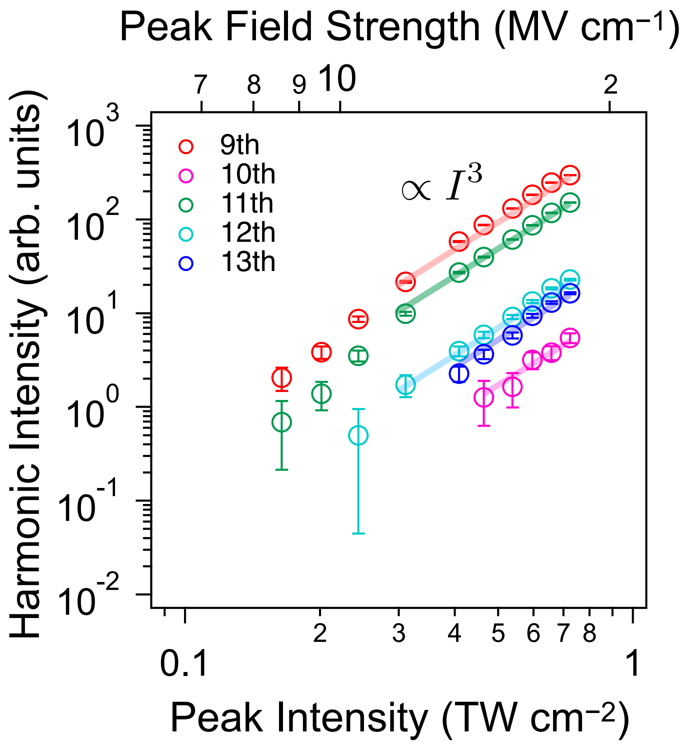

Supplementary Figure 4 Excitation power dependence of the high harmonic intensity from monolayer MoS_2. Intensities of harmonic radiation of various orders from monolayer MoS_2 as a function of peak intensity of the excitation. The error bars represent the standard deviations given

by the multiple measurements. The solid lines are guides for the eye, indicating the harmonic intensities are proportional to I^3.

IV. Supplementary Note 3: Band structure of monolayer MoS_2 determined using tight-binding model

The band structure of the monolayer MoS_2 used for the semi-classical calculation shown in Fig. 4 (Main Text) and Supplementary Figures 7(b) and 7(c) is given by the tight binding model developed in Suppl. Refs. [1] and [2]. The valence and conduction bands have the following equations.

$$\varepsilon_{\pm}(\mathbf{K}) = \frac{\varepsilon_{\mathrm{m}}(\mathbf{K})+\varepsilon_{\mathrm{s}}(\mathbf{K})}{2} \pm \frac{1}{2}\sqrt{[\varepsilon_{\mathrm{m}}(\mathbf{K}) - \varepsilon_{\mathrm{s}}(\mathbf{K})]^2 + 4|t_{\mathrm{sm}}(\mathbf{K})|^2}, \tag{1}$$

where

$$\varepsilon_{\ell}(\mathbf{K}) = \varepsilon_{\ell} + \sum_{\delta>0} 2t_{\ell\ell}(\boldsymbol{\delta}) \cos(\mathbf{K}\cdot\boldsymbol{\delta}), \quad \ell = \mathrm{s}, \mathrm{m}, \tag{2}$$

$$|t_{\mathrm{sm}}(\mathbf{K})| = t_{\mathrm{sm}}\sqrt{3 + 2\cos K_x a + 4\cos\frac{K_x a}{2}\cos\frac{\sqrt{3}K_y a}{2}}. \tag{3}$$

The parameters given in Suppl. Ref. [1] are summarized in **Supplementary Table 1**.

Supplementary Table 1 The list of the band parameters. The parameters used for the band calculation taken from Suppl. Ref. [1].

δ	a	$\sqrt{3}a$	$2a$	$\sqrt{7}a$	$3a$	$2\sqrt{3}a$	$\sqrt{13}a$	$4a$
t_{ss}/meV	45	15	60	0	5	−35	5	15
t_{mm}/meV	20	100	10	−5	−5	10	0	−5

$\varepsilon_{\mathrm{m}} = 1.15$ eV, $\varepsilon_{\mathrm{s}} = -1.7$ eV, $t_{\mathrm{sm}} = 0.3$ eV

By using the obtained tight-binding band structure, we also calculated the group velocity $\dot{\mathbf{r}} = \hbar^{-1}\partial\varepsilon(\mathbf{k})/\partial\mathbf{k}$, where $\varepsilon(\mathbf{k})$ is the electron's energy, $\mathbf{r}$ is the carrier's position and $\mathbf{k}$ its wave vector, as shown in Supplementary Figure 5. This result was used in the calculation of the motion of the electrons and holes in Fig. 4 in the main text. Supplementary Figure 6 shows the calculated joint density of states along the $K - \Gamma - K'$ trajectory in the reciprocal space. It shows divergence features at 1.8, 2.8, 2.9, and 3.3 eV. The peak at 1.8 eV is derived from the bandgap at K and K' points, and that at 3.3 eV is derived from the Γ point. The peaks at 2.8 and 2.9 eV corresponds to the band nesting region where the optical absorption and high harmonic intensity have the resonant enhancement (Fig. 2 in the main text).

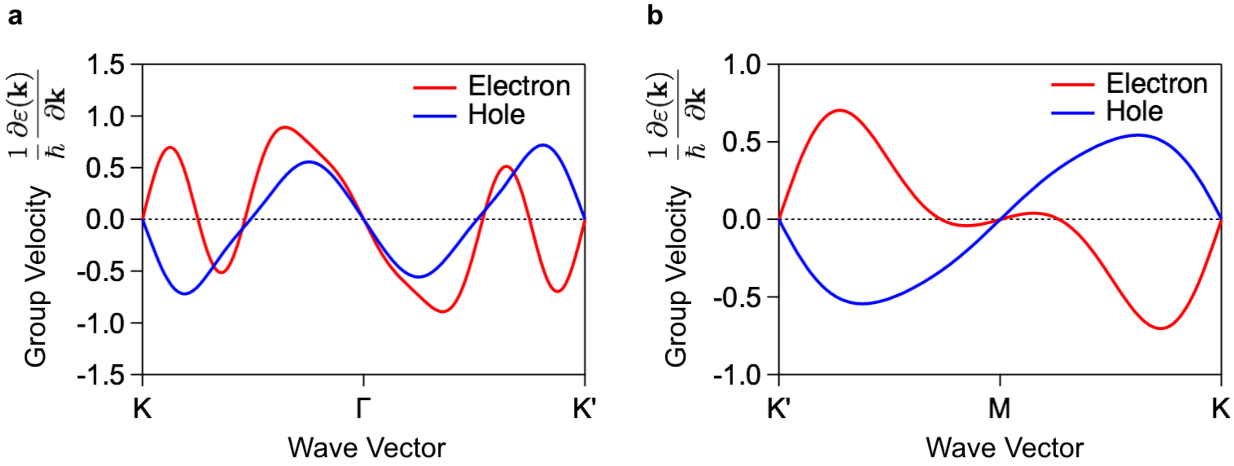

Supplementary Figure 5 Calculated group velocity of monolayer MoS_2. Group velocity of electrons and holes in monolayer MoS_2 for **a** $K - \Gamma - K'$ and **b** $K - \mathrm{M} - K'$.

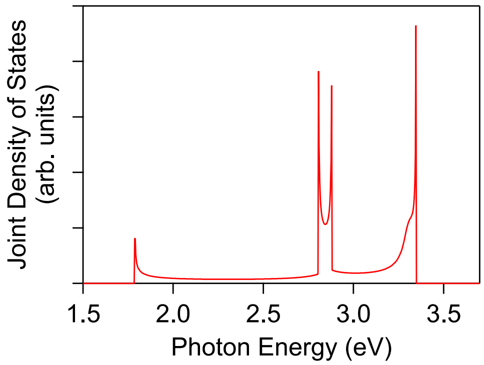

Supplementary Figure 6 Calculated joint density of states of monolayer MoS_2. Joint density of states of monolayer MoS_2 calculated along the $K - \Gamma - K'$ trajectory in the reciprocal space.

V. Supplementary Note 4: Symmetry analysis of polarization selection rules

In the main text, the polarization selection rules of HHG with the zigzag excitation are discussed. Here, we derive the polarization selection rule from the microscopic model which describes the three step HHG mechanism in solids. Using the formulation discussed in Suppl. Ref. [3], the interband polarization density $\mathbf{p}(t)$ can be written in the length gauge as follows:

$$\mathbf{p}(t) = \frac{\mathrm{i}}{\hbar}\int_{\mathrm{BZ}} \mathbf{d}^2\mathbf{k}\, \boldsymbol{\mu}^*(\mathbf{k}) \frac{\mathrm{i}}{\hbar}\int_{-\infty}^{t} dt' \boldsymbol{\mu}\big(\mathbf{k}(t,t')\big) \cdot \mathbf{E}_{\mathrm{MID}}(t') \exp\left(-\mathrm{i}\int_{t'}^{t} dt''\, \varepsilon_{\mathrm{g}}\big(\mathbf{k}(t,t'')\big)/\hbar\right) + \mathrm{c.c.}\,, \tag{4}$$

$$\mathbf{k}(t,t') = \mathbf{k} + \frac{e}{\hbar}\mathbf{A}(t') - \frac{e}{\hbar}\mathbf{A}(t). \tag{5}$$

Here, $\mathbf{k}$ is the crystal momentum, $\boldsymbol{\mu}$ is the transition dipole moment, ε_{g} is the transition energy from the valence to the conduction band, and $\mathbf{A}(t)$ is the vector potential of the driving field satisfying $\mathbf{E}_{\mathrm{MID}}(t) = -\partial\mathbf{A}(t)/\partial t$. The equation (4) describes the induced dipole moment for

HHG including electron-hole pair creation through the tunneling process and electron-hole motion due to the driving field. For simplicity, we do not account for the spin-orbit coupling in this model. In order to derive the selection rule for HHG, we divide $\mathbf{p}(t)$ into two terms given by

$$\mathbf{p}(t) = p_+(t)\boldsymbol{\sigma}^+ + p_-(t)\boldsymbol{\sigma}^-, \tag{6}$$

$$p_\pm(t) = \frac{\mathrm{i}}{\hbar}\int_{\mathrm{BZ}} \mathbf{d^2k}\, \mu_\pm^*(\mathbf{k}) \int_{-\infty}^{t} dt' \boldsymbol{\mu}\big(\mathbf{k}(t,t')\big) \cdot \mathbf{E}_{\mathrm{MID}}(t') \exp\left(-\mathrm{i}\int_{t'}^{t} dt''\, \varepsilon_{\mathrm{g}}\big(\mathbf{k}(t,t'')\big)/\hbar\right) + \mathrm{c.\,c.}, \tag{7}$$

Then, let us consider continuous-wave electric field along zigzag direction, where $\mathbf{E}_{\mathrm{zigzag}}^{\mathrm{MID}}(t) = E_x \cos(\Omega t)\,(\boldsymbol{\sigma}^+ + \boldsymbol{\sigma}^-)/\sqrt{2}$, and the temporal-translation of the interband polarization $\mathbf{p}(t + T/2)$ ($T = 2\pi/\Omega$) is given by

$$p_\pm\left(t + \frac{T}{2}\right)$$
$$= -\frac{\mathrm{i}}{\hbar}\int_{\mathrm{BZ}} \mathbf{d^2k}\, \mu_\pm^*(-k_x, k_y) \int_{-\infty}^{t} dt' \mu_x\big(-k_x(t,t'), k_y\big) E_x \cos(\Omega t') \exp\left(-\mathrm{i}\int_{t'}^{t} dt''\, \varepsilon_{\mathrm{g}}\big(-k_x(t,t''), k_y\big)/\hbar\right)$$
$$+\mathrm{c.\,c.}, \tag{8}$$

$$k_i(t,t') = k_i + \frac{e}{\hbar}A_i(t') - \frac{e}{\hbar}A_i(t). \quad (i = x \text{ and } y) \tag{9}$$

$$\mu_x = (\mu_+ + \mu_-)/\sqrt{2}. \tag{10}$$

Since the electronic system in TMDs without the driving field $\mathbf{E}_{\mathrm{zigzag}}^{\mathrm{MID}}(t)$ has a mirror symmetry plane normal to the x-axis (zigzag axis), the transition energy ε_{g} and the dipole moment $\boldsymbol{\mu}$ satisfy

$$\varepsilon_{\mathrm{g}}(-k_x, k_y) = \varepsilon_{\mathrm{g}}(k_x, k_y), \tag{11}$$

$$\mu_{\pm}(-k_x, k_y) = -\mu_{\mp}(k_x, k_y). \tag{12}$$

These conditions do not supply any relation between $p_+(t)$ and $p_-(t)$ but give a relation between $\mathbf{p}(t + T/2)$ and $\mathbf{p}(t)$ as follows:

$$p_{\pm}\left(t + \frac{T}{2}\right)$$

$$= -\frac{\mathrm{i}}{\hbar}\int_{\mathrm{BZ}} \mathbf{d^2k}\, \mu_{\mp}^{*}(k_x, k_y) \int_{-\infty}^{t} dt' \mu_x\big(k_x(t,t'), k_y\big) E_x \cos(\Omega t') \exp\left(-\mathrm{i}\int_{t'}^{t} dt''\, \varepsilon_{\mathrm{g}}\big(k_x(t,t''), k_y\big)/\hbar\right)$$

$$+ \mathrm{c.c.}$$

$$= -p_{\mp}(\mathrm{t}). \tag{13}$$

Since electric field of high-harmonics is approximately given by $\mathbf{E}^{\mathrm{HHG}}(t) \propto d^2\mathbf{p}(t)/dt^2$, we can obtain Eq. (2) in the main text ($E_{\pm}^{\mathrm{HHG}}(t) = -E_{\mp}^{\mathrm{HHG}}(t + \frac{T}{2})$), resulting in parallel (x-polarized) odd-order harmonics and perpendicular (y-polarized) even-harmonics that we observed in the experiments.

Next, we consider the polarization of HHG with the armchair excitation. The electric field of the excitation is described with the polarization of the armchair direction (y axis) of the mid-infrared excitation, as $\mathbf{E}_{\mathrm{armchair}}^{\mathrm{MID}}(t) = E_y \cos(\Omega t)\, (\boldsymbol{\sigma}^{+} - \boldsymbol{\sigma}^{-})/\sqrt{2}\mathrm{i}$. In contrast to the zigzag excitation case, one can find a simple relation between $p_+(t)$ and $p_-(t)$ since the driving field is within the mirror symmetry plain. Using the equations (11) and (12), the relation between $p_+(t)$ and $p_-(t)$ is given by

$p_{\pm}(t)$

$$= \frac{\mathrm{i}}{\hbar}\int_{\mathrm{BZ}} \mathbf{d^2k}\, \mu_{\pm}^{*}(-k_x, k_y) \int_{-\infty}^{t} dt' \mu_y\left(-k_x, k_y(t,t')\right) E_y \cos(\Omega t') \exp\left(-\mathrm{i}\int_{t'}^{t} dt''\, \varepsilon_{\mathrm{g}}\left(-k_x, k_y(t,t'')\right)/\hbar\right)$$

$+\,\mathrm{c.\,c.}$

$$= -\frac{\mathrm{i}}{\hbar}\int_{\mathrm{BZ}} \mathbf{d^2k}\, \mu_{\mp}^{*}(k_x, k_y) \int_{-\infty}^{t} dt' \mu_y\left(k_x, k_y(t,t')\right) E_y \cos(\Omega t') \exp\left(-\mathrm{i}\int_{t'}^{t} dt''\, \varepsilon_{\mathrm{g}}\left(k_x, k_y(t,t'')\right)/\hbar\right)$$

$+\mathrm{c.\,c.}$

$$= -p_{\mp}(t), \tag{14}$$

$$\mu_y = (\mu_+ - \mu_-)/\sqrt{2}\mathrm{i}. \tag{15}$$

As a result, one can deduce the rules:

$$E_{\pm}^{\mathrm{HHG}}(t) = -E_{\mp}^{\mathrm{HHG}}(t) \tag{16}$$

Equation (S16) leads to the following equation:

$$E_{x}^{\mathrm{HHG}}(t) = 0, \tag{17}$$

This means that both even- and odd-order HHG emission appear with the parallel polarization (*y*-polarized) in the case of armchair excitation.

We briefly touch the reason why we can obtain the parallel-polarized even-harmonics although the motions of electron-hole pairs in the ranges $0 < t < T/2$ and $T/2 < t < T$ are symmetric in the case of armchair excitation. The temporal-translation of the interband polarization $\mathbf{p}(t + T/2)$ is given by

$$p_\pm\left(t+\frac{T}{2}\right)=$$

$$-\frac{\mathrm{i}}{\hbar}\int_{\mathrm{BZ}}\mathbf{d^2k}\,\mu_\pm^*\left(k_x,-k_y\right)\int_{-\infty}^{t}dt'\mu_y\left(k_x,-k_y(t,t')\right)E_y\cos(\Omega t')\exp\left(-\mathrm{i}\int_{t'}^{t}dt''\,\varepsilon_{\mathrm{g}}\left(k_x,-k_y(t,t'')\right)/\hbar\right)+$$

$$\mathrm{c.c.} \tag{18}$$

Since electronic system in TMDs has the time reversal symmetry, transition energy ε_{g} and dipole moment $\boldsymbol{\mu}$ satisfy

$$\varepsilon_{\mathrm{g}}(-\mathbf{k})=\varepsilon_{\mathrm{g}}(\mathbf{k}), \tag{19}$$

$$\mu_\pm(-\mathbf{k})=\mu_\mp^*(\mathbf{k}). \tag{20}$$

By combining equations (11) and (12) from the mirror symmetry, one can obtain

$$\varepsilon_{\mathrm{g}}\left(k_x,-k_y\right)=\varepsilon_{\mathrm{g}}\left(k_x,k_y\right), \tag{21}$$

$$\mu_\pm\left(k_x,-k_y\right)=-\mu_\pm^*\left(k_x,k_y\right). \tag{22}$$

By using these relations, $\mathbf{p}(t+T/2)$ becomes

$$p_\pm\left(t+\frac{T}{2}\right)$$

$$=\frac{\mathrm{i}}{\hbar}\int_{\mathrm{BZ}}\mathbf{d^2k}\,\mu_\pm\left(k_x,k_y\right)\int_{-\infty}^{t}dt'\mu_y^*\left(k_x,k_y(t,t')\right)E_y\cos(\Omega t')\exp\left(-\mathrm{i}\int_{t'}^{t}dt''\,\varepsilon_{\mathrm{g}}\left(k_x,k_y(t,t'')\right)/\hbar\right)$$

$$+\,\mathrm{c.c.}$$

$$=\frac{\mathrm{i}}{\hbar}\int_{\mathrm{BZ}}\mathbf{d^2k}\,\left|\mu_\pm(\mathbf{k})\right|\int_{-\infty}^{t}dt'\left|\mu_y\left(\mathbf{k}(t,t')\right)\right|\mathrm{e}^{\mathrm{i}\left\{\theta_\pm(\mathbf{k})-\theta_y\left(\mathbf{k}(t,t')\right)\right\}}E_y\cos(\Omega t')\exp\left(-\mathrm{i}\int_{t'}^{t}dt''\,\varepsilon_{\mathrm{g}}\left(\mathbf{k}(t,t'')\right)/\hbar\right)$$

$$+\mathrm{c.c.}, \tag{23}$$

$\mu_i = |\mu_i| \mathrm{e}^{\mathrm{i}\theta_i}$. (24)

On the other hand, $\mathbf{p}(t)$ can be written by

$p_\pm(t) =$

$$\frac{\mathrm{i}}{\hbar}\int_{\mathrm{BZ}} \mathbf{d^2k}\, |\mu_\pm(\mathbf{k})| \int_{-\infty}^{t} dt' \left|\mu_y\big(\mathbf{k}(t,t')\big)\right| \mathrm{e}^{-\mathrm{i}\left\{\theta_\pm(\mathbf{k})-\theta_y\left(\mathbf{k}(t,t')\right)\right\}} E_y \cos(\Omega t') \exp\left(-\mathrm{i}\int_{t'}^{t} dt''\, \varepsilon_{\mathrm{g}}\big(\mathbf{k}(t,t'')\big)/\hbar\right) +$$

$\mathrm{c.c.}$. (25)

From the equations (23) and (24), it is clear that $\mathbf{p}(t + T/2)$ and $\mathbf{p}(t)$ have a difference in the phase of the complex dipole moment such as $\left\{\theta_\pm(\mathbf{k}) - \theta_y\big(\mathbf{k}(t,t')\big)\right\}$, permitting the even-orders of HHG in the parallel polarization with the driving field, even though motions of the electron-hole pair are symmetric in the case of armchair excitation .

VI. Supplementary Note 5: Calculated dynamics of electrons and holes under mid-infrared excitation with the armchair polarization

In the main text, we used the extended three-step model to examine the dynamics of electrons and holes with the zigzag-polarization excitation. Here, we discuss the dynamics for the armchair-polarization excitation. Supplementary Figure 7a shows the group velocity map of the conduction band electrons in momentum space, as calculated with the tight binding model. With the armchair excitation (in the k_y direction represented by the orange arrows), the wave packets of Bloch electrons are accelerated not only in the y direction but also in the x direction in real space, whereas they are accelerated only in the x direction with the zigzag excitation (in the k_x direction represented by the green arrows). We regard a collision of an electron and hole as a pair whose separation is

smaller than 0.05 a. Here, we assume that these electron-hole pairs are generated at the K and K' points at $t = 0$, at which the positive peak of the incident light field occurs. We calculated the dynamics of a hole generated at $(x, y) = (0,0)$ and several electrons generated at 60 neighboring atomic sites in addition to the atomic site of the hole. We made a 2D-search for collisions between the electrons and the hole with the limited collision time of a half cycle of the incident light field $(0 < t < T/2)$. The two circles in Supplementary Figures 7b and 7c show the recombined electron-hole pairs in momentum space. Initial positions of the electron and the holes are depicted in Supplementary Figure 7d for two possible recombination paths. Even with the armchair polarization, the electrons and holes recombine and HHG is emitted. It is clear that the collision dynamics of the electrons and holes generated at the K points are equivalent to those at the K' points as is clearly shown in Supplementary Figures 7b and 7c. This situation is reproduced by the theoretical model described in the Supplementary Note 4. However, we should also pay attention to the result that the phases of induced polarizations are different by π as shown in Eqn. (14) in Supplementary Note 4. These results clearly predict that no HHG is observed with perpendicular polarization (zigzag) in the case of the armchair excitation, which is experimentally confirmed in Supplementary Figure 3.

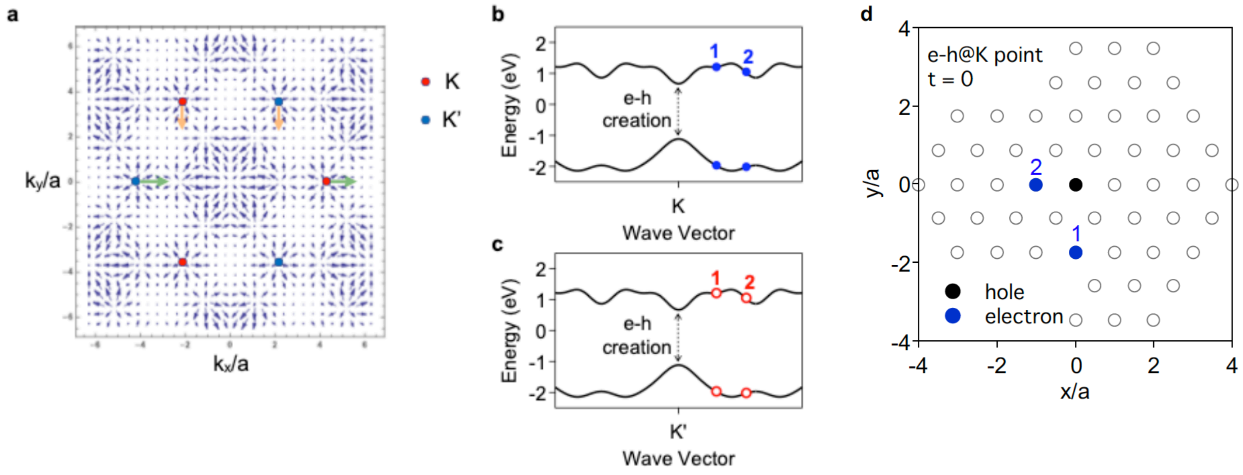

Supplementary Figure 7 Calculation of dynamics for the armchair polarization. a Group velocity map of conduction band electrons calculated using tight-binding model. Orange (green) arrows represent the acceleration direction in the armchair (zigzag) polarization. **b, c** Recombined electron-hole pairs generated at K and K' in momentum space along armchair direction. The recombined electron-hole pairs created at the K (K') point are represented as blue solid (red open) circles. The labels 1 and 2 indicate the two possible recombination paths at $t = 0.09T$ and $t = 0.26T$, respectively. **d** Initial positions of the electrons and the hole (fixed at the origin) for two possible recombination paths.

Supplementary References

[1] Rukelj, Z., Strkalj, A., and Despoja V., Optical absorption and transmission in a molybdenum disulfide monolayer, Phys. Rev. B **94**, 115428 (2016).

[2] Kupcic, I., Damping effects in doped graphene: The relaxation-time approximation, Phys. Rev. B **90**, 205426 (2014).

[3] Vampa, G., McDonald, C. R., Orlando, G., Klug, D. D., Corkum, P. B., and Brabec, T., Theoretical analysis of high-harmonic generation in solids, Phys. Rev. Lett. **113**, 073901 (2014).
